# The complete chloroplast genome of *Chrysoglossum ornatum* (Epidendroideae, Orchidaceae) and its phylogenetic analysis

**DOI:** 10.1080/23802359.2023.2296920

**Published:** 2024-01-03

**Authors:** Cheng-Jiang Tan, Rong-Rong Yan, Ping Yu, Guang-Qin Lu, Wei Wu, Guo-Xiong Hu

**Affiliations:** a Management Department of Maolan National Nature Reserve, Libo, China; b College of Life Sciences, Guizhou University, Guiyang, China

**Keywords:** *Chrysoglossum*, Collabieae, plastid genome, phylogenetic relationship

## Abstract

*Chrysoglossum ornatum* Blume, the type species of *Chrysoglossum* Blume, belongs to the tribe Collabieae of the subfamily Epidendroideae of Orchidaceae. In this study, we sequenced, assembled, and analyzed the complete chloroplast genome of *C. ornatum*. The result showed that the complete chloroplast genome of *C. ornatum* was 158,175 bp in size, consisting of a large single-copy (LSC) region of 87,235 bp, a small single-copy (SSC) region of 18,384 bp, and a pair of inverted repeats (IRs) of 26,278 bp. The chloroplast genome encoded 113 unique genes, comprising 80 protein-coding genes, 29 tRNA genes, and four rRNA genes. Phylogenetic analysis inferred from the complete chloroplast genome indicated that *Chrysoglossum* was closely related to *Collabium* Blume. This study provides genomic resources helpful for further phylogenetic and biodiversity research on *Chrysoglossum*.

## Introduction

Orchidaceae is one of the largest families of flowering plants, with approximately 736 genera and 28,000 species (Christenhusz and Byng 2016). Molecular phylogenetic studies have recognized five monophyletic subfamilies within Orchidaceae, namely Apostasioideae, Vanilloideae, Cypripedioideae, Orchidoideae, and Epidendroideae (Chase et al. 2015). *Chrysoglossum* Blume, a small genus with four species (*Chrysoglossum assamicum* Hook. f., *Chrysoglossum ensigerum* W. Burgh & de Vogel, *Chrysoglossum ornatum* Blume, and *Chrysoglossum reticulatum* Carr), belongs to the tribe Collabieae of the subfamily Epidendroideae of Orchidaceae and mainly ranges from tropical Asia to New Guinea and the Pacific islands (Xiang et al. 2014; Zhou et al. 2023). *Chrysoglossum* is morphologically similar to the genus *Collabium* Blume and was once treated as a synonym of *Collabium* (Seidenfaden 1983). Phylogenetic analyses based on four plastid markers demonstrated that *Collabium* and *Chrysoglossum* should be maintained as two distinct genera (Xiang et al. 2014). However, the phylogenetic resolution among closely related species was very low (Xiang et al. 2014).

Compared with nuclear and mitochondrial genomes, the chloroplast genome has great advantages for genetic and phylogenetic analyses, owing to its stable genetic structure, relatively small size, and moderate nucleotide substitutions (Daniell et al. 2016). However, to date, the chloroplast genomes of *Chrysoglossum* are still lacking. In this study, we sequenced, assembled, and analyzed the complete chloroplast genome of *Chrysoglossum ornatum*, which will contribute to further phylogenetic and biodiversity studies of *Chrysoglossum*.

## Materials and methods

### Plant material, DNA extraction, and sequencing

**Figure 1.**
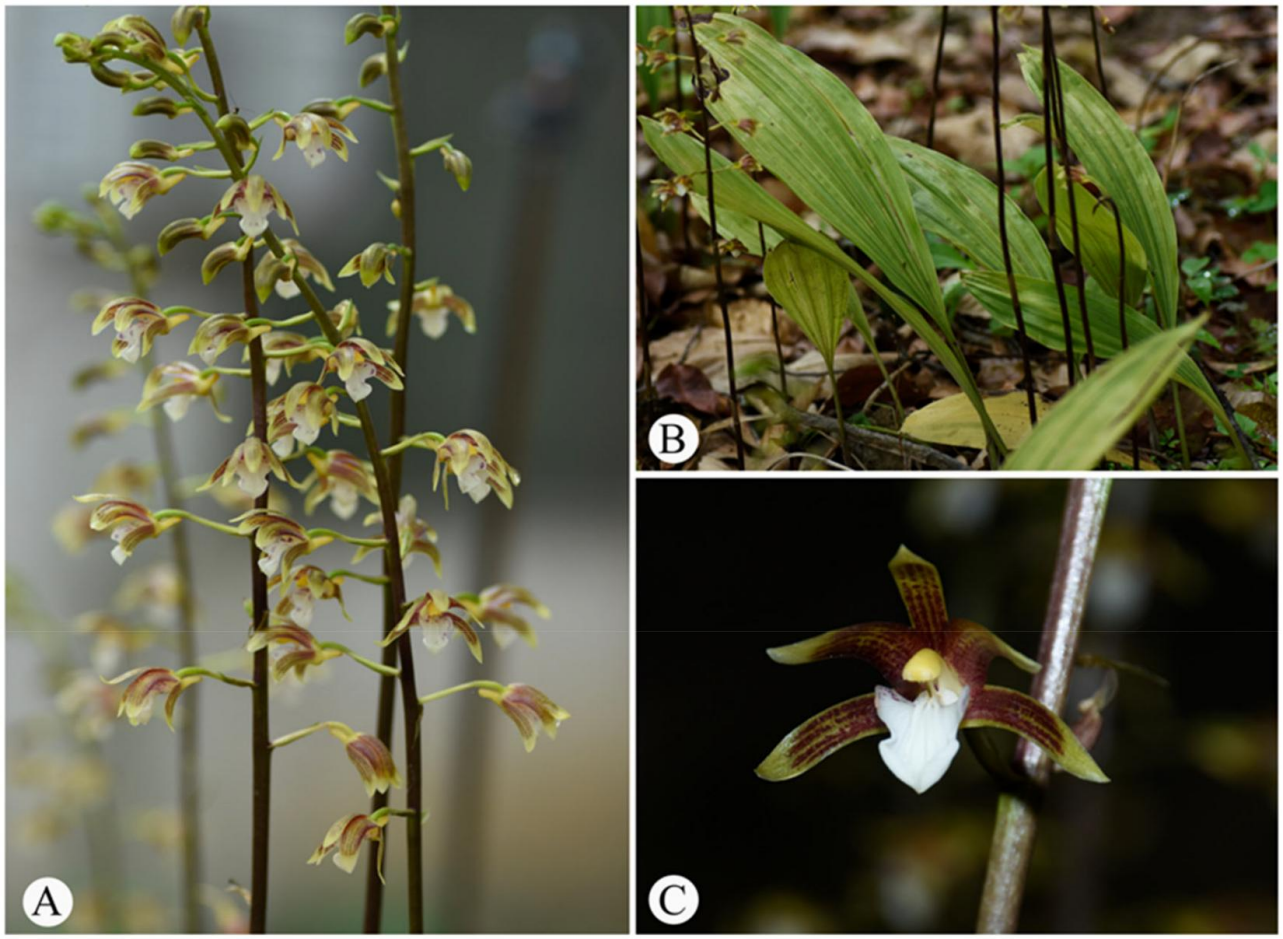
Photos of *Chrysoglossum ornatum*. (A) Inflorescences; (B) plants; (C) flower. The photographs were taken by Wei Wu.

*Chrysoglossum ornatum* was collected from the Management Department of Maolan National Nature Reserve, Libo, Guizhou, China (107°52′51.23″E, 25°24′48.96″N) (Figure 1). The voucher specimen was deposited in the Herbarium of the Natural Museum of Guizhou University (GACP) with voucher number 2023Co001 (contact: Guoxiong Hu, gxhu@gzu.edu.cn). Total genomic DNA was isolated from the silica-dried leaves using the modified CTAB method (Doyle and Doyle 1987). DNA integrity and concentration were assessed with agarose gel electrophoresis and a NanoDrop 2000 spectrophotometer. The genomic DNA was fragmented randomly to construct shotgun libraries for paired-end (150 bp) sequencing using the Illumina Hiseq 2500 platform by BGI Technology Service Co., Ltd. (Wuhan, China). Low-quality reads were filtered with SOAPnuke v.2.0 (Chen et al. 2018), generating 7,201,363,200 clean bases.

### Genome assembly and annotation

The chloroplast genome was assembled using GetOrganelle v1.7.5 (Jin et al. 2020) with clean data, following default settings. To verify the correctness of assembly, we checked the genome structure in Bandage v0.8.1 (Wick et al. 2015) (Figure S1) and generated a coverage plot by matplotlib v.3.7.0 (https://matplotlib.org/) (Figure S2). The web server CPGAVAS2 (Shi et al. 2019) was used to annotate the chloroplast genome with *Collabium formosanum* (NC_065862) as the reference. Then, we manually calibrated the start and stop codons in Geneious v9.0.2 software (Kearse et al. 2012). The genome map was drawn using CPGView (Liu et al. 2023).

### Phylogenetic analyses

Phylogenetic tree was constructed based on the complete chloroplast genome using maximum-likelihood (ML) and Bayesian inference (BI) methods in CIPRES Science Gateway (https://www.phylo.org/). All sequences were aligned using MAFFT v.7.388 with auto strategy (Katoh and Standley 2013). The GTRGAMMA model was employed to infer the ML tree, with 1000 bootstrap (BS) replicates. The best-fitting model of BI analysis was selected according to Akaike information criterion (AIC) (David and Buckley 2004). The BI tree was constructed under the GTR + F + I + G4 model using Markov Chain Monte Carlo (MCMC) algorithm, with 2,000,000 generations independently and every 1000 generations for tree sampling. Finally, we edited these results in the iTOL (Letunic and Bork 2007).

## Results

**Figure 2.**
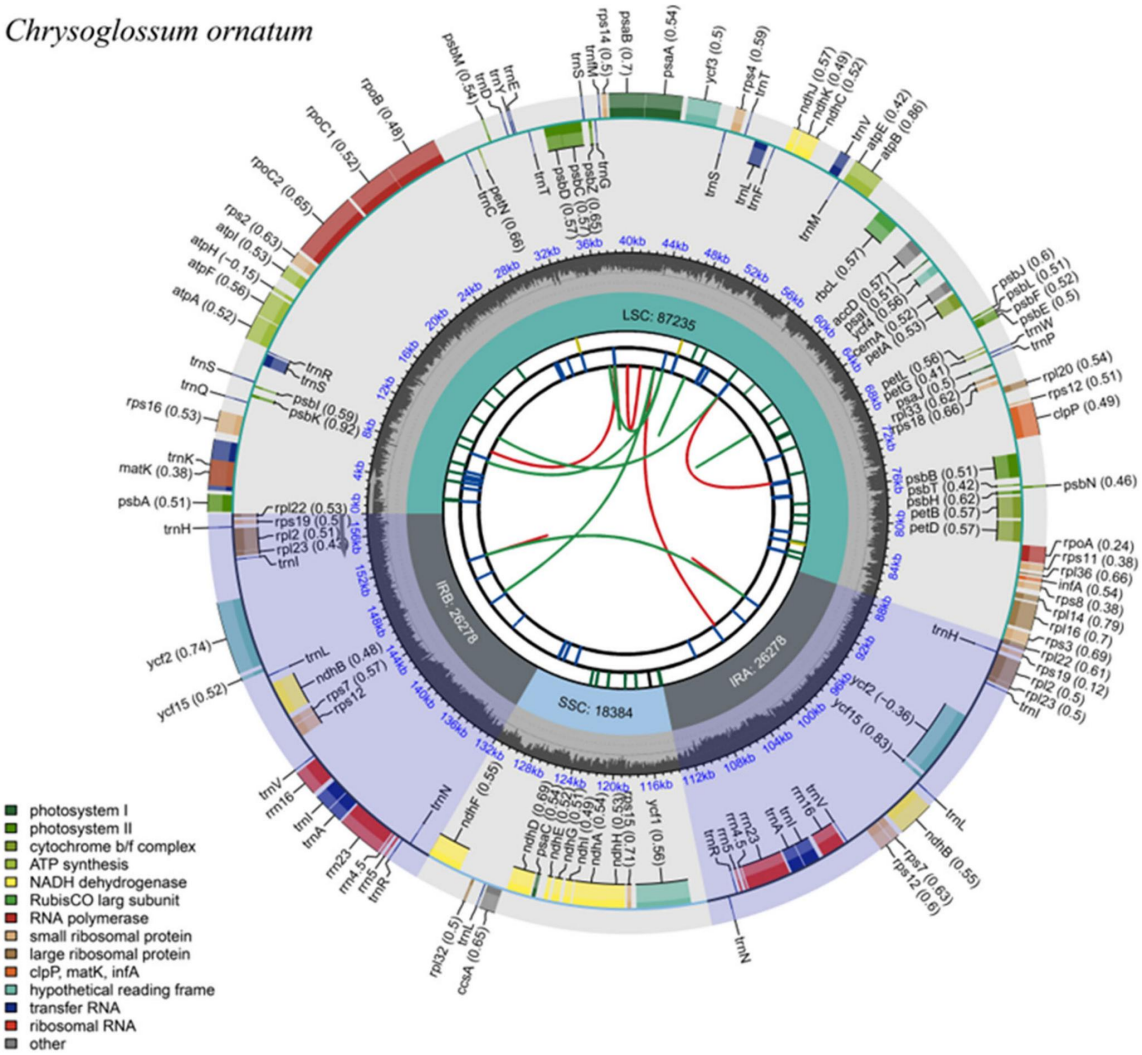
The chloroplast genome map of *Chrysoglossum ornatum*. From the center outward, the map consists of six rings. The first circle represents the forward and reverse repeats connected with red and green arcs, respectively. The second circle shows the tandem repeats marked. The third circle displays the microsatellite sequences. The fourth circle indicates the sizes of feature regions, including a large single-copy (LSC), a small single-copy (SSC), and two inverted repeats (IRa and IRb). The fifth circle exhibits the GC content. The sixth circle presents the genes with different functions.

The complete chloroplast genome of *Chrysoglossum ornatum* exhibited a typical quadripartite structure with 158,175 bp in length, comprising a large single-copy (LSC) region of 87,235 bp, a small single-copy (SSC) region of 18,384 bp, and two inverted repeats (IRs) of 26,278 bp (Figure 2). The GC content was uneven in *C. ornatum*, with 43.28%, 34.82%, and 30.22% in the IR, LSC, and SSC regions, respectively. A total of 113 unique genes were annotated, consisting of 80 protein-coding genes, 29 tRNA genes, and four rRNA genes. Among these genes, 22 genes were duplicated in the IR region, including nine protein-coding genes (*ndhB*, *rpl2, rpl22, rpl23*, *rps7*, *rps12*, *rps19*, *ycf15*, *ycf2*), nine tRNA genes (*trnA-UGC*, *trnH-GUG*, *trnI-CAU*, *trnI-GAU*, *trnL-CAA*, *trnN-GUU*, *trnR-ACG*, *trnS-GGA*, *trnV-GAC*), and four rRNA genes (*rrn16*, *rrn23*, *rrn4.5*, *rrn5*). In addition, 19 genes with intron were observed, of which *clpP*, *rps12*, *ycf1*, and *ycf3* genes contained two introns and others (*atpF*, *ndhA*, *ndhB*, *petB*, *petD*, *rpl16*, *rpl2*, *rps16*, *rpoC1*, *trnA-UGC*, *trnI-GAU*, *trnK-UUU*, *trnL-UAA*, *trnS-CGA*, *trnV-UAC*) possessed a single intron. The gene *rps12* with the 5′ end of exon located in the LSC and 3′ end of exon located in the IR was identified as a trans-splicing gene (Figure S3).

**Figure 3.**
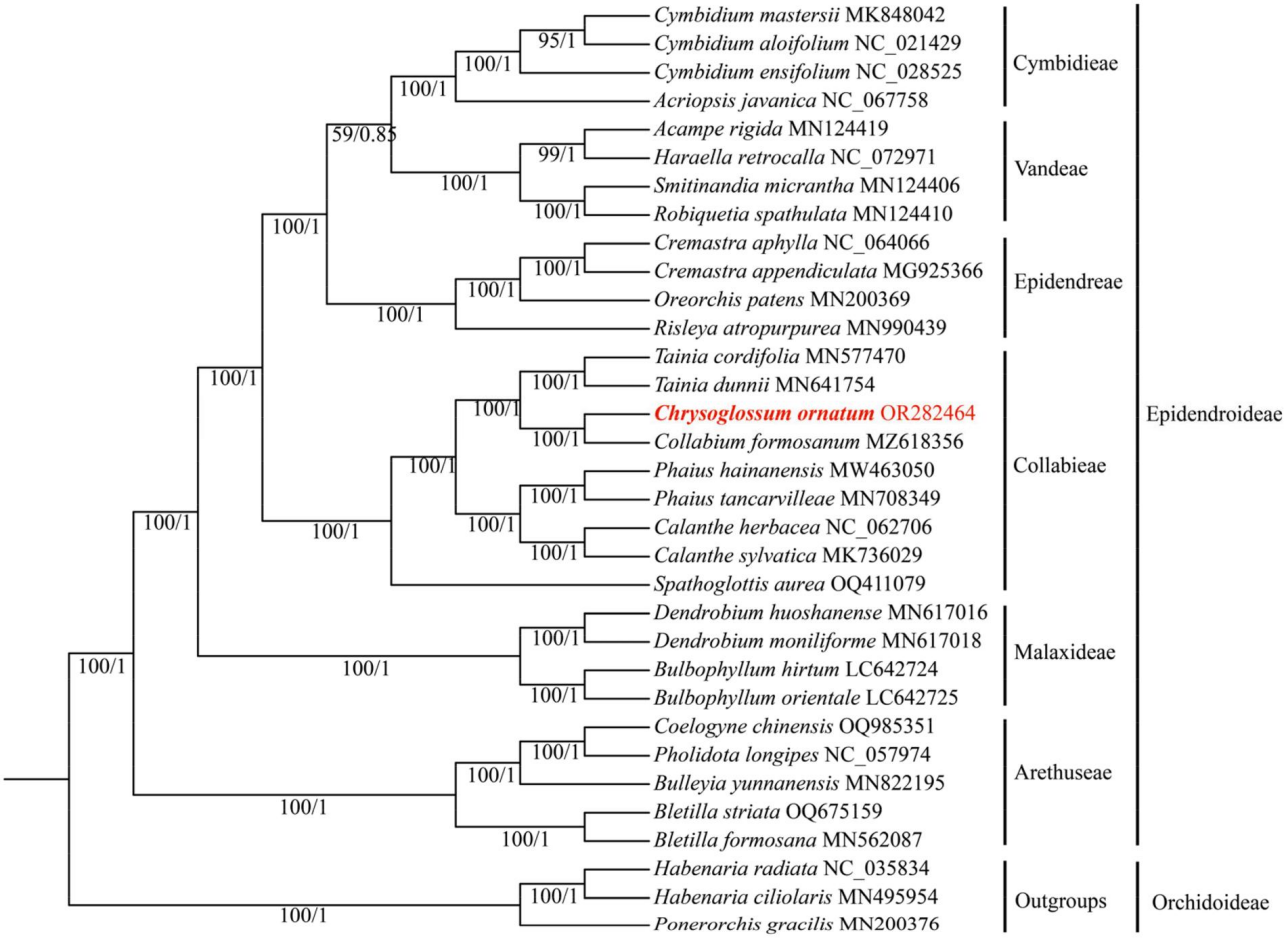
Phylogenetic tree inferred from the complete chloroplast genomes comprising 33 species from Orchidaceae. The ML bootstrap (BS) and BI posterior probability (PP) that supported each node are shown under the branches. The following sequences were used: *Acampe rigida* (Liu et al. 2020), *Bletilla formosana* (Wu et al. 2019), *Bletilla striata* (Feng et al. 2019), *Bulbophyllum hirtum* (Yang et al. 2022), *Bulbophyllum Orientale* (Yang et al. 2022), *Bulleyia yunnanensis* (Ai et al. 2020), *Calanthe sylvatica* (Miao et al. 2019), *Cremastra appendiculata* (Mao et al. 2018), *Cymbidium aloifolium* (Chen et al. 2020), *Cymbidium ensifolium* (Jiang et al. 2019), *Cymbidium mastersii* (Zheng et al. 2019), *Habenaria ciliolaris* (Chen et al. 2019), *Oreorchis patens* (Kim et al. 2020), *Ponerorchis gracilis* (Kim et al. 2020), *Robiquetia spathulata* (Liu et al. 2020), *Smitinandia micrantha* (Liu et al. 2020), *Tainia cordifolia* (Zheng et al. 2019), and *Tainia dunnii* (Xie et al. 2019).

In total, 33 species were included in phylogenetic analyses, of which 30 species were from the subfamily Epidendroideae, with the other three species from the subfamily Orchidoideae as the outgroups (Figure 3). The phylogenetic trees inferred from ML and BI yielded identical topologies. As a result, only the ML tree was presented with ML bootstrap support (BS) and BI posterior probability (PP) values indicated. Phylogenomic analysis showed that *Chrysoglossum* was sister to *Collabium* with a full support value (BS = 100, PP = 1.00).

## Discussion and conclusions

Similar to most angiosperms (Daniell et al. 2016), the complete chloroplast genome of *Chrysoglossum ornatum* displayed a typical quadripartite structure. Generally, the size of the chloroplast genome in the land plants ranges from 120 to 160 kb encoding 110 to 130 unique genes (Green 2011). *Chrysoglossum ornatum* showed relative conservation in genome size and gene content. In Flora Reipublicae Popularis Sinicae, *Chrysoglossum* and *Collabium* were placed in the subtribe Collabiinae, and *Risleya* was placed in the subtribe Risleyinae, all of which belonged to the tribe Epidendreae. Using four DNA markers, however, *Chrysoglossum* was sister to *Risleya* and clustered together with *Collabium*, in which the three genera were retreated into the tribe Collabieae (Xiang et al. 2014). The treatment was followed by Chase et al. (2015). Nevertheless, Li et al. (2020) based on the complete plastid data suggested that the genus *Risleya* should be placed in the tribe Epidendreae. In this study, phylogenetic analyses demonstrated that *Chrysoglossum* was closely related to *Collabium* with strong support (BS = 100, PP = 1.00) and also supported the treatment of *Risleya* from Collabieae to Epidendreae. It is worth mentioning that phylogenetic analysis based on the complete chloroplast genome generated a higher resolution than that inferred from a few DNA markers. Overall, our study provides a molecular resource for phylogenetic and evolutionary studies of *Chrysoglossum.*

## Supplementary Material

Supplemental Material

## Data Availability

The chloroplast genome of *Chrysoglossum ornatum* was deposited in GenBank of NCBI at https://www.ncbi.nlm.nih.gov/ under the accession number OR282464. The associated BioProject, BioSample, and SRA numbers are PRJNA995913, SAMN36510063, and SRR25319442, respectively.
