# Supplementary material for: The complete chloroplast genome of Chrysoglossum ornatum (Epidendroideae, Orchidaceae) and its phylogenetic analysis
Source: Mitochondrial DNA B Resour. 2024 Jan 3;9(1):24–8. doi: 10.1080/23802359.2023.2296920 (PMC10769113; doi:10.1080/23802359.2023.2296920)
Supplement: Supplemental Material [file TMDN_A_2296920_SM8742.docx]

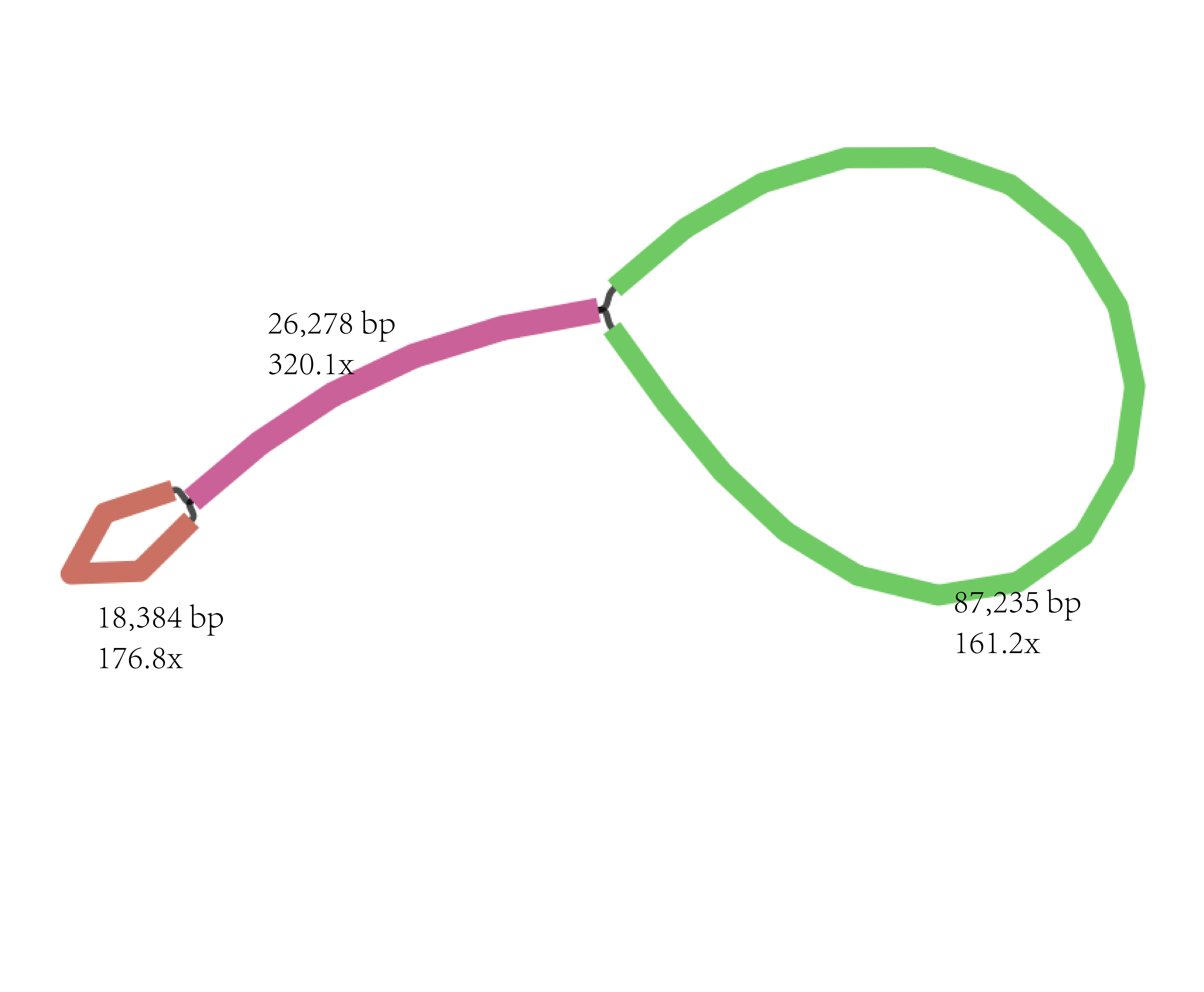


Supplementary Figure 1. The quadripartite structure and coverage depth in Bandage.


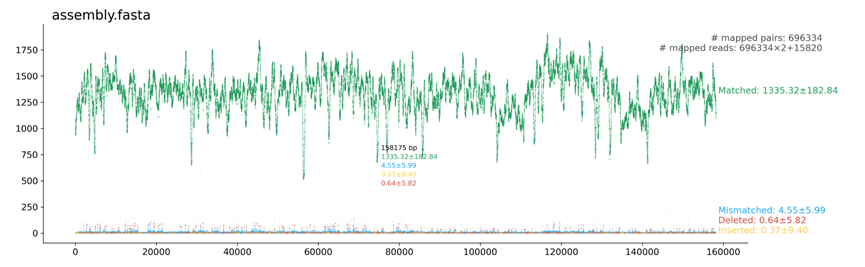


Supplementary Figure 2. The coverage plot across the assembled chloroplast genome of *Chrysoglossum ornatum*.


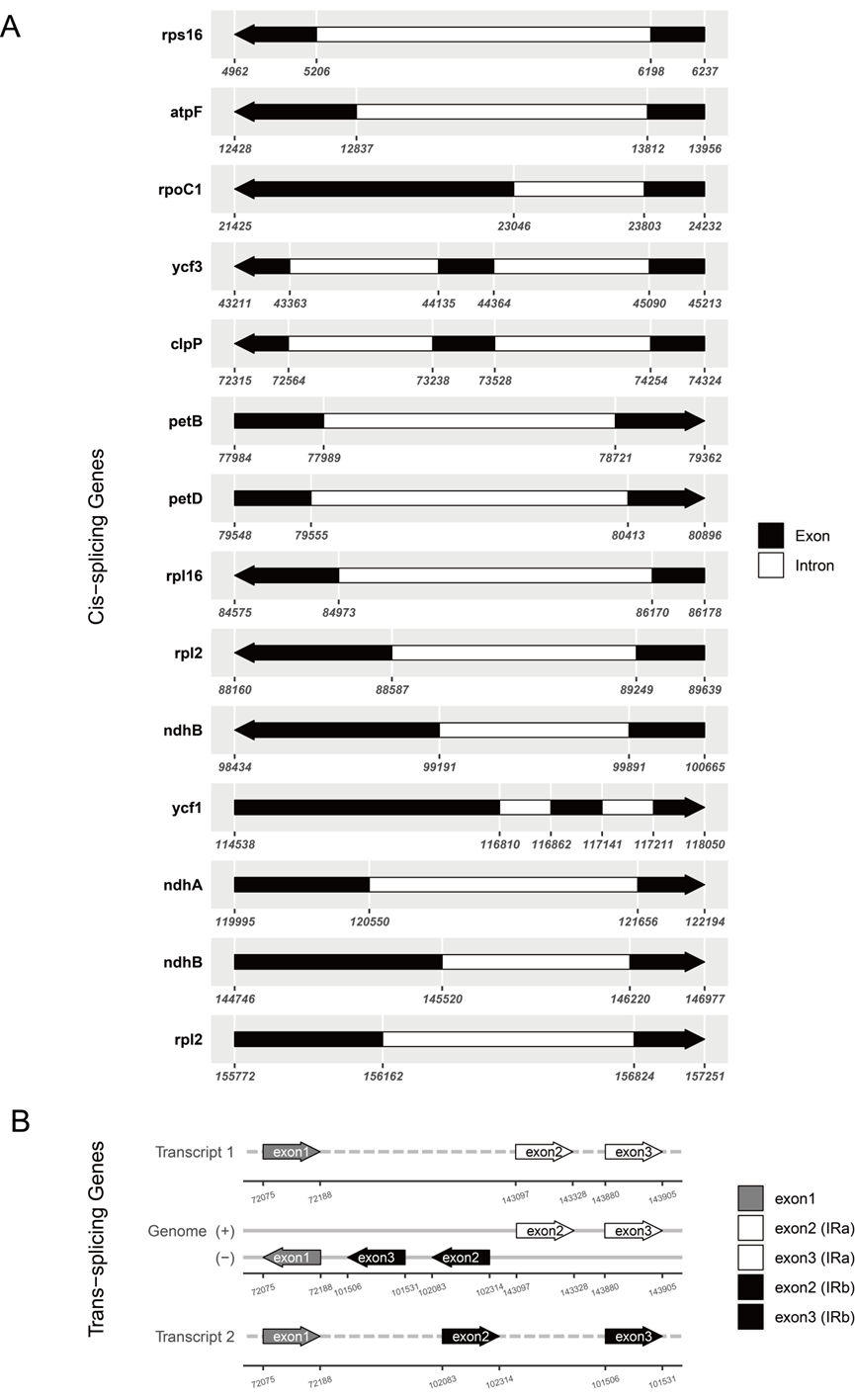


Supplementary Figure 3. Schematic map of the cis-splicing and trans-splicing genes. A, cis-splicing genes; B, trans-splicing genes.
